# Characterization of a Multidrug-Resistant, Novel *Bacteroides* Genomospecies

**DOI:** 10.3201/eid2101.140662

**Published:** 2015-01

**Authors:** Stephen J. Salipante, Aley Kalapila, Paul S. Pottinger, Daniel R. Hoogestraat, Lisa Cummings, Jeffrey S. Duchin, Dhruba J. Sengupta, Steven A. Pergam, Brad T. Cookson, Susan M. Butler-Wu

**Affiliations:** University of Washington, Seattle, Washington, USA (S.J. Salipante, A. Kalapila, P.S. Pottinger, D.R. Hoogestraat, L. Cummings, J.S. Duchin, D.J. Sengupta, S.A. Pergam, B.T. Cookson, S.M. Butler-Wu);; Fred Hutchinson Cancer Research Center, Seattle (A. Kalapila, S.A. Pergam)

**Keywords:** genome, Bacteroides, genomospecies, multidrug resistance, metronidazole-resistance, intra-abdominal infection, bacteria, novel

## Abstract

Metronidazole- and carbapenem-resistant *Bacteroides fragilis* are rare in the United States. We isolated a multidrug-resistant anaerobe from the bloodstream and intraabdominal abscesses of a patient who had traveled to India. Whole-genome sequencing identified the organism as a novel *Bacteroides* genomospecies. Physicians should be aware of the possibility for concomitant carbapenem- and metronidazole-resistant *Bacteroides* infections.

We previously reported a 2013 case of intraabdominal abscesses and bacteremia caused by a multidrug-resistant anaerobe identified as *Bacteroides fragilis* (*1*). In brief, unremitting abdominal pain developed in a 71-year-old man who had been traveling in India for 1 month. The man was hospitalized locally and subsequently received a diagnosis of metastatic colon adenocarcinoma. He returned to Seattle, Washington, USA, for treatment consisting of 5 cycles of chemotherapy, followed by right hemicolectomy and right hepatectomy. On postoperative day 4, the patient showed marked leukocytosis, and abdominal abscesses were noted on computed tomographic scan images. Cultured percutaneous drainage fluid grew *Escherichia coli* that was resistant to ampicillin, trimethoprim/sulfamethoxazole, and fluoroquinolones. Therapy was then limited to ceftriaxone, and the patient’s leukocyte count continued to rise and fever returned. Blood cultures grew anaerobic gram-negative rods identified as *B. fragilis* by MALDI-TOF (matrix-assisted laser desorption ionization-time of flight) mass spectrometry and 16S rRNA sequencing. New rim-enhancing fluid collections in the abdomen and pelvis were noted on computed tomographic scan images, and percutaneous drainage fluid from these collections grew 3+ (moderate) quantities of *B. fragilis*. Isolates from blood culture and abscess fluid were resistant to multiple classes of antimicrobial drugs, including metronidazole and imipenem (Table). The abscesses ultimately resolved after treatment for 60 days with linezolid and empiric ertapenem.

**Table:** Antimicrobial susceptibility results for a novel *Bacteroides* genomospecies isolated from the bloodstream and intraabdominal abscesses of a patient with colon cancer, 2013

Antimicrobial drug	MIC, μg/mL*
Ampicillin/sulbactam	>256/128
Cefotetan	64
Clindamycin	>256
Imipenem	>32†
Linezolid	2
Metronidazole	>256†
Minocycline	4
Moxifloxacin	>32
Piperacillin/tazobactam	>256
Synercid	>32
Tetracycline	16
Ticarcillin/clavulanic acid	>256/2
Tigecycline	1

*Antimicrobial susceptibility testing performed by using E-test (Technical Appendix). †MICs confirmed as >64 μg/mL by macrobroth dilution.

## The Study

To better characterize the patient’s clinical isolate, we subjected the organism to whole-genome sequencing by using the MiSeq platform (Illumina, San Diego, CA, USA). In brief, DNA was digested by using NEBNext dsDNA Fragmentase and then end-repaired and A-tailed by using *E. coli* DNA Polymerase I, T4 PNK, and Taq DNA Polymerase (all from New England Biolabs, Ipswich, MA, USA). Annealed Y adaptors (5′-(PO4-) GATCGGAAGAGCGGTTCAGCAGGAATGCCGAG-3′ and 5′-ACACTCTTTCCCTACACGACGCTCTTCCGATCT-3′) were ligated by using T4 DNA Ligase in Rapid Ligation Buffer (Enzymatics, Beverly, MA, USA). The library was PCR-amplified with KAPA HiFi HotStart ReadyMix (Kapa Biosystems, Wilmington, MA, USA) by using primer 1 (5′-AATGATACGGCGACCACCGAGATCTACACTCTTTCCCTACACGACGC-3′) and primer 2 (5′-CAAGCAGAAGACGGCATACGAGATCAAGGTCACGGTCTCGGCATTCCTGCTGAACCG-3′). For sequencing, 250-bp paired-end reads were used with a custom index primer (5′-AGATCGGAAGAGCGGTTCAGCAGGAATGCCGAGACCG-3′); sequencing was performed to an estimated coverage of 61× per base. Oligonucleotides were synthesized by Integrated DNA Technologies (Coralville, IA, USA). De novo genome assembly was performed by using the ABySS v1.3.5 assembler (*2*); gene prediction and annotation, using the RAST server v4.0 (*3*); and comparative genomic analyses, using jSpecies v1.2.1 (*4*). The assembly was visualized by using BRIG 0.95 (*5*).

Initial comparison of the clinical isolate with the 3 completed *B. fragilis* reference genomes (638R, YCH46, NCTC 9343) showed a high degree of sequence divergence (Figure). We expanded our analysis to other sequenced *Bacteroides* species and observed similar results (Figure). An average nucleotide identity by BLAST (http://blast.ncbi.nlm.nih.gov/Blast.cgi) (ANIb) analysis (*4*) was performed (Technical Appendix Table 1). Of note, pairwise ANIb values of <95% have been used as the cutoff for circumscribing species (*4*). The clinical isolate demonstrated pairwise ANIb values of 86.28%–86.54% against *B. fragilis* reference strains and even lower values compared with other *Bacteroides* species. In contrast, divergence among the 3 *B. fragilis* reference strains averaged 98.64% identity. These data are consistent with the conclusion that the isolate represents a genomospecies distinct from *B. fragilis*. This relationship was confirmed by using a BLAST search of the assembly against the nonredundant NCBI (National Center for Biotechnology Information) sequence database that contains published bacterial sequences; confirmation of the relationship indicated that the organism most closely resembles *B. fragilis* 638R and is not better classified as an alternative species already present in GenBank.

**Figure:**
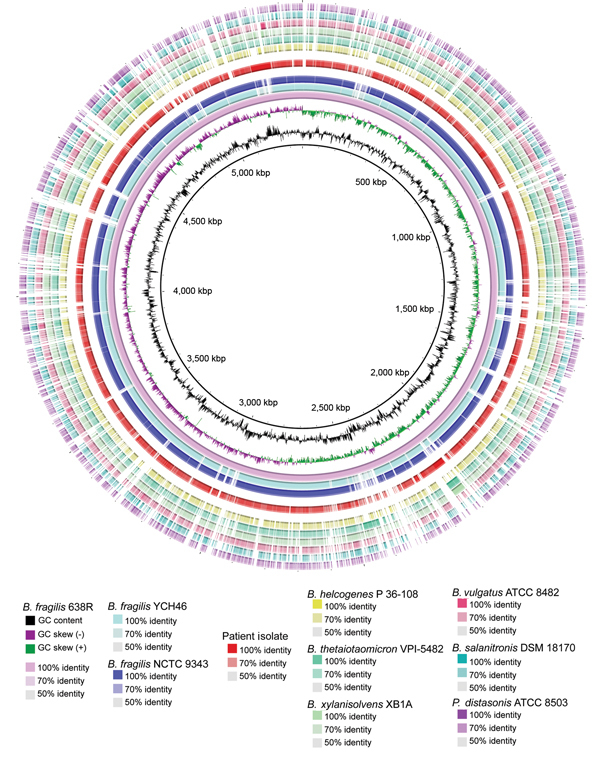
Characterization of circular plot of genome diversity between the clinical isolate of a multidrug-resistant, novel *Bacteroides* genomospecies and other *Bacteroides* spp. isolates. Reading from the center outwards, the map, GC content, and GC skew of the *B. fragilis* reference strain 638R are depicted. The white and colored regions of the following outer rings indicate regions absent and present, respectively, in genomes of the indicated organism compared with the genome of *B. fragilis* reference strain 638R. Intensity of coloration is proportional to the degree of sequence identity relative to the reference genome. The innermost 3 rings indicate the 3 *B. fragilis* reference genomes. The genome of the clinical isolate, separated from other rings by white space, follows. Non-*fragilis Bacteroides* species and a *Parabacteroides* species comprise the outermost rings. ATCC, American Type Culture Collection; DSM, Deutsche Sammlung von Mikroorganismen; NCTC, National Collection of Type Cultures.

The clinical isolate contained an estimated 5.50 Mbp of DNA, ≈20% more than sequenced *B. fragilis* reference strains, and 43.81% GC content. The shotgun sequence encodes 5,053 predicted genes, including 1,479 hypothetical proteins without inferred function. This number is significantly (1-tailed z-score = 1) larger than the average predicted gene content of *B. fragilis* reference genomes (average of 4,760 predicted genes), although we cannot rule out minor contributions from plasmid DNA. Of 1,696 *B. fragilis* core genes (those present in all *B. fragilis* reference strains), 1,508 (88.9%) were present in the clinical isolate.

We explored the basis of the isolate’s antimicrobial resistance by performing a BLAST search of the assembly against a database of previously described known factors (*6*) (Technical Appendix Table 2). Carbapenem resistance in *B. fragilis* has been shown to result from up-regulation of the *cfiA* metallo-β-lactamase (*7*), and we identified homologs of 2 β-lactamases, *cfxA and cfiA13*, in the clinical isolate (the latter of which had an upstream insertion sequence). Although the exact mechanism of metronidazole resistance is unknown, it has been attributed to chromosomally or plasmid-encoded nitroimidazole resistance (*nim*) genes encoding nitroimidazole reductase (*8*–*10*). Although we did not detect homologs of canonical genes *nimA*–*J*, 2 putative nitroimidazole resistance genes were identifiable on the basis of functional annotation (*10*). We also detected *ermF* (macrolide resistance) and *tetQ* genes (tetracycline resistance). The isolate had a substitution in *gyrA* (Ser82 to Phe) known to confer resistance to fluoroquinolones (*11*). The draft genome (GenBank accession no. JANI00000000) and sequence reads (Sequence Read Archive accession no. SRP045260) are publicly accessible.

## Conclusions

Members of the *Bacteroides* genus constitute a large fraction of the human gut microbiome and are important opportunistic pathogens that can cause a variety of serious infections. Metronidazole is thought to be almost universally effective against the species: only 1 of 1,957 *B. fragilis* clinical isolates collected across the United States during 2006–2009 was resistant to metronidazole (*12*). Similarly, previous studies have shown a prevalence of only ≈1% of carbapenem-resistant *B. fragilis* (*13*), making this a favored second-line treatment (*7*). Nevertheless, there are limited but increasing reports of concomitant metronidazole and carbapenem resistance (*14*), and such resistance poses a threat to current treatment algorithms.

Genomic sequencing revealed the isolate in this study to be a genomospecies related to, but distinct from, *B. fragilis*. Techniques widely accepted as highly accurate for bacterial identification (16S rRNA sequencing and MALDI-TOF mass spectrometry) incorrectly identified this organism as *B. fragilis* (*1*). To determine whether previous multidrug-resistant isolates identified as *B. fragilis* might instead represent this novel genomospecies, we compared ANIb values for other isolates with those of the clinical isolate in our study. ANIb values indicate that 2 of the multidrug-resistant *B. fragilis* isolates sequenced in an earlier report (*10*) actually appear to be members of the genomospecies reported here (Technical Appendix Table 3). Furthermore, these data highlight the potential for whole-genome sequencing to improve diagnostic accuracy in microbial identification and isolate characterization. Although we detected several antimicrobial drug–resistance factors in this isolate, alternative mechanisms of resistance to carbapenems and metronidazole, including non-*cfiA*–mediated carbapenem resistance and overexpression of efflux genes, respectively, have been also described and may contribute to the high levels of resistance observed with this isolate.

The increasing prevalence of carbapenem-resistant *Enterobacteriaceae* worldwide poses a major public health concern, often requiring use of more toxic “last-line agents,” such as colistin and polymyxin B. Although rare thus far, multidrug-resistant *Bacteroides* infections present a similar challenge to the treating physician and have the potential to become increasingly common in the future. Thus, providers should be aware of the possibility for concomitant carbapenem and metronidazole resistance in these organisms. Future studies will be required to determine what proportion of multidrug-resistant isolates can be ascribed to the novel genomospecies of *Bacteroides* reported here and whether this particular genomospecies offers any opportunities for targeted therapies.

Technical Appendix Values for the clinical isolate and other *Bacteroides* species and antibiotic resistance genes in clinical isolate.
