# Supplementary material for: Characterization of a Multidrug-Resistant, Novel Bacteroides Genomospecies
Source: Emerg Infect Dis. 2015 Jan;21(1):95–8. doi: 10.3201/eid2101.140662 (PMC4285247; doi:10.3201/eid2101.140662)
Supplement: Technical Appendix — Values for the clinical isolate and other Bacteroides species and antibiotic resistance genes in clinical isolate. [file 14-0662-Techapp-s1.pdf]

Article DOI: http://dx.doi.org/10.3201/eid2101.140662

Characterization of a Multidrug-Resistant, Novel *Bacteroides* Genomospecies

Technical Appendix

Technical Appendix Table 1. ANIb values for the clinical isolate and other *Bacteroides* species*

	B. fragilis strain									
Species	638R	YCH46	NCTC 9343	Clinical isolate	*B. helcogenes* P 36–108	*B. thetaiotaomicron* VPI-5482	*B. xylanisolvens* XB1A	*B. vulgatus* ATCC 8482	*B. salanitronis* DSM 18170	*Parabacteroides distasonis* ATCC 8503
B. fragilis 638R	**100**	**98.58†**	**98.56**	86.63	72.57	75.28	75.3	71.1	69.49	67.43
B.fragilis YCH46	**98.76†**	**100**	**98.6**	86.64	72.52	74.42	74.97	70.89	70.54	67.47
B. fragilis NCTC 9343	**98.77**	**98.59**	**100**	86.64	72.72	74.28	74.22	70.82	69.53	67.36
Clinical Isolate	86.54	86.43	86.28	**100**	72.39	75.16	75.63	72.03	70.63	68.02
B. helcogenes P 36–108	72.65	72.67	72.86	72.69	**100**	72.7	72.67	70.76	69.98	67.43
B. thetaiotaomicron VPI-5482	74.9	74.04	73.97	74.93	72.52	**100**	80.42	71.19	69.5	68.14
B. xylanisolvens XB1A	74.9	74.63	74.11	75.1	72.4	80.7	**100**	71.25	69.91	67.4
B. vulgatus ATCC 8482	71.01	71.06	70.86	73	71.05	72.06	71.49	**100**	71.18	68.62
B.salanitronis DSM 18170	69.75	70.66	69.73	70.81	70.16	69.99	70.16	70.97	**100**	68.07
Parabacteroides distasonis ATCC 8503	67.72	67.45	67.74	68.05	67.41	68.53	67.41	68.32	67.77	**100**

*Bold font indicates ANIb values >95%, ANIb, average nucleotide identity by BLAST.
†Pairwise ANIb values demonstrate minor differences due to inherent stochasticity in the calculation (*1*).

Technical appendix Table 2. Antibiotic resistance genes in clinical isolate

Resistance_type	contig_id	feature_id	start	stop	strand	RAST-assigned function	figfam_identification	nucleotide_sequence	aa_sequence
putative nitroimidazole resistance	427	fig|6666666.52503.peg.184	47597	48070	+	Pyridoxamine 5'-phosphate oxida	FIG01045148	atgaagacagtaatcattgaaaacaaagagcaggtagaagaaattatctcccgttgcgatatttgctttgtcggtatcaccgacctggaaggcaacccttatgtgatccccatgaacttcggctatcaggatggagtgatctatctgcattccggccccacaggcagcagcatcgatatgctggcaaaaaacaaccgtgtctgcatcactttcagtgtagaccacgaattggtattccagcaccccaaggtggcatgcagttatcggatgcgggccaaaagtgtgatttgcagaggacgcgtaaactttatcgaagacctggaagaaaaacgtgaagcgctcaatatattgatgcgccattatagcagccgggaatttgtatactcagatccggcagtgagaaatgtaaaaatctgggagataccgattgaaaacgttacggcaaaagagtatgcggtgccgcatacgaaataa	MKTVIIENKEQVEEIISRCDICFVGITDLEGNPYVIPMNFGYQDGVIYLHSGPTGSSIDMLAKNNRVCITFSVDHELVFQHPKVACSYRMRAKSVICRGRVNFIEDLEEKREALNILMRHYSSREFVYSDPAVRNVKIWEIPIENVTAKEYAVPHTK
putative nitroimidazole resistance	500	fig|6666666.52503.peg.3895	62001	61498	-	Pyridoxamine 5'-phosphate oxida	FIG01045148	atggaatatatcaacgatttaatccgacggaaggaccgccttttatcggaagacgaagcaatgagtttacttgaaaatggagaatacggagtgttatctatttcttcacctgaagaaggtgtttatggcattcccattaattatgtatgggataaacaacagtctatctattttcattgtgctcccgaagggcgtaagctttgtatacttcaagactgtaatcaggcatcgttttgcgtggtaggacgcacgcgcgttgtttccaatcaatttagtaccgagtatgaaagtatcgtgattaacgggactatgatttgtcagttagaagaaactgagaagcgaaaggcattggaacttctgcttgataaatattctccggaagataaaaaggtggggttgaagtatatggaaaagtcttttcaccgtacccacgtcttgaaacttgttattaaaacaatatccggtaagtgtaagaaaatgaatcagccctgtccggattattaa	MEYINDLIRRKDRLLSEDEAMSLLENGEYGVLSISSPEEGVYGIPINYVWDKQQSIYFHCAPEGRKLCILQDCNQASFCVVGRTRVVSNQFSTEYESIVINGTMICQLEETEKRKALELLLDKYSPEDKKVGLKYMEKSFHRTHVLKLVIKTISGKCKKMNQPCPDY
cfiA14 Beta-lactamase	439	fig|6666666.52503.peg.820	32936	32187	-	Beta-lactamase	FIG00021206	atgaaaacagtatttatccttatctccatgcttttccctgtcgcagttatggcacagaaaagcgtaaaaatatccgatgatatcagtatcacccaactctcggacaaagtgtacacttatgtatccctcgccgaaatcgaaggatggggtatggtaccttccaacgggatgattgttatcaacaaccaccaggcagcgttgctggacacaccgatcaatgacgcacaaacggaaatactggtcaactgggtgacagactctttgcatgccaaagtcaccacgtttatcccgaaccactggcacggcgattgtattggcggactgggttacctgcaaaggaaaggtgtccaatcatacgcgaaccagatgacgatagacctcgccaaggaaaaagggttgcccgtaccggaacatggattcaccgattcactgaccgtcagcttggacggcatgcctctccaatgctattatttaggaggcggacatgcgaccgacaatatcgtggtttggctgccgacagagaatatcctttttggcggatgtatgcttaaagacaaccaggcgacaagcatcggcaatatctcggacgcggacgtgacggcatggccgaaaactctcgataaggtaaaagccaagttcccctcggcccgctacgtcgtgcccggacatggcgactatggcggaaccgaactgatagagcataccaagcagatcgtgaaccaatatatagaaagcacctcaaaaccatag	MKTVFILISMLFPVAVMAQKSVKISDDISITQLSDKVYTYVSLAEIEGWGMVPSNGMIVINNHQAALLDTPINDAQTEILVNWVTDSLHAKVTTFIPNHWHGDCIGGLGYLQRKGVQSYANQMTIDLAKEKGLPVPEHGFTDSLTVSLDGMPLQCYYLGGGHATDNIVVWLPTENILFGGCMLKDNQATSIGNISDADVTAWPKTLDKVKAKFPSARYVVPGHGDYGGTELIEHTKQIVNQYIESTSKP
TetQ Tetracycline resistance protein	461	fig|6666666.52503.peg.1666	15624	13699	-	Tetracycline resistance protein Te	FIG01956083	atgaatattataaatttaggaattcttgctcacattgatgcaggaaaaacttccgtaaccgagaatctgctgtttgccagtggagcaacggaaaagtgcggccgtgtggataatggtgacaccataacggactctatggatatagagaaacgtagaggaattactgtccgggcttctacgacatctattatctggaatggagtgaaatgcaatatcattgacactccgggacacatggattttattgcggaagtggagcggacattcaaaatgcttgatggagcagtcctcatcttatccgcaaaggaaggcatacaagcgcagacaaagttgctgttcagtactttacaaaagctgcaaatcccgacaattatatttatcaataagattgaccgtgccggtgtgaatttggagcgtttgtatatggatataaaaacaaatctgtcgcaagatgtcctgtttatgcaaactgttgtcgatggatcggtttatccggtttgctcccaaacatatataaaggaagaatacaaagaatttgtatgcaaccatgacgacgatatattagaacgatatttggcggatagcgaaatttcaccggctgattattggaatacgataatcgctcttgtggcaaaagccaaagtctatccggtgctacatggatcagcaatgttcaatatcggtatcaatgagttgttggacgccatttcttcttttatacttcctccggcatcagtctcaaacagactttcagcttatctctataagatagagcatgaccccaaagggcataaaagaagttttcttaaaataattgacggaagtctgagacttcgagacgttgtaagaatcaacgattcggaaaaattcatcaagattaaaaatctaaagactatttatcagggcagagagataaatgttgatgaagtgggtgccaatgatatcgcgattgtagaagatatagaagattttcgaatcggagattatttaggtgctaaaccttgtttgattcaaggattatctcatcagcatcccgctctcaaatcctccgtccggccaaataagcccgaagagagaagcaaggtgatatccgctctgaatacattgtggattgaagacccgtctttgtccttttccataaactcatatagtgatgaattggaaatctcgttatatggtttgacccaaaaggaaatcatacagacattgctggaagaacgattttccgtaaaggtccattttgatgagatcaagactatctacaaagaacgacctataaaaaaggtcaataagattattcagatcgaagtaccacccaacccttactgggccacaatagggctgactcttgaacccttaccgttaggggcagggttgcaaatcgaaagtgacatctcctatggttatctgaaccattcttttcaaaatgccgtttttgaagggattcgtatgtcttgccaatctggtttacatggatgggaagtgacagatctgaaagtaacttttactcaagccgagtattatagcccggtaagtacacctgctgatttcagacagctgaccccttatgtcttcaggctggctttgcaacagtcaggtgtggacattctcgaaccgatgctctgttttgagttgcagataccccaagtagcgagttccaaagctattacagatttgcaaaaactgatgtctgagattgaagacatcagttgtaataatgagtggtgtcatattaaagggaaagttccattaaatacaagtaaagactatgcctcagaagtaagttcgtacactaagggcttaggcattttttatggttaagccatgtgggtatcaaataacaaaagacggttattctgataatatccgcatgaacgaaaaagataaacttttattcatgttccaaaaatcaatgtcattaaaataa	MNIINLGILAHIDAGKTSVTENLLFASGATEKCGRVDNGDTITDSMDIEKRRGITVRASTTSIIWNGVKCNIIDTPGHMDFIAEVERTFKMLDGAVLILSAKEGIQAQTKLLFSTLQKLQIPTIIFINKIDRAGVNLERLYMDIKTNLSQDVLFMQTVVDGSVYPVCSQTYIKEEYKEFVCNHDDDILERYLADSEISPADYWNTIIALVAKAKVYPVLHGSAMFNIGINELLDAISSFILPPASVSNRLSAYLYKIEHDPKGHKRSFLKIIDGSLRLRDVVRINDSEKFIKIKNLKTIYQGREINVDEVGANDIAIVEDIEDFRIGDYLGAKPCLIQGLSHQHPALKSSVRPNKPEERSKVISALNTLWIEDPSLSFSINSYSDELEISLYGLTQKEIIQTLLEERFSVKVHFDEIKTIYKERPIKKVNKIIQIEVPPNPYWATIGLTLEPLPLGAGLQIESDISYGYLNHSFQNAVFEGIRMSCQSGLHGWEVTDLKVTFTQAEYYSPVSTPADFRQLTPYVFRLALQQSGVDILEPMLCFELQIPQVASSKAITDLQKLMSEIEDISCNNEWCHIKGKVPLNTSKDYASEVSSYTKGLGIFMVKPCGYQITKDGYSDNIRMNEKDKLLFMFQKSMSLK
cfxA Beta-lactamase	487	fig|6666666.52503.peg.3116	79479	80444	+	Beta-lactamase (EC 3.5.2.6)	FIG00000747	atgaaaaaaagtagaagaaaacgccttgtaattttgtgtatagctttagtttgtatcatagttttagtttttttattgttctctaaatcaacttctaataatagtacaaatccaccttgacagatgttttgactgatagcatttctcggatagtatcagcttgtcctggtgaaattggagtggcaattattattaataacacagatacagttaaagttaatgataagagtgtatatcctatgatgagtgtatttaaggttcatcaggcattagctctttgcaatgattttgataacaaagggatttcacttgataccttagtaaagatagataggaatagacttgattcaaagacttggagtcctatgatgaaagattattccgagttggttatatcattaaccgtgagagatttacttcgttatactattgctcaaagtgataataatgcaagcaatctgatgtttaaagatatggtcaatgttgctcaaactgacagttttatagccacacttattcctcgttcaagttttcagatagcttatacagaagaggaaatgtcggctgaccacgatagggcttactttaattatacatctcctcttggtgccgcaatgttgatgaaccgtttgtttaccgaaagtattgtcagtggtgagaagcaaagtttcattaagaatacattaaaagaatgtgtgacaggtacagatagaatagtcgctccgcttcttgataaagaaagggtttctattgcacataagacgggttctggtgatgtcaatgaaaatggtattcttgcagctcacaatgatgttgcctatatatgcctgcctaataatgtctgctatactttagcgatatttgttaaggatttcaagggtaatgaatcacaagcatcacaatatgttgcgcatatatcagaggtcgtatattctttattaatacaaaattcggcaattccttaa	MKKSRRKRLVILCIALVCIIVLVFLLFSKSTSNNSTNPPLTDVLTDSISRIVSACPGEIGVAIIINNTDTVKVNDKSVYPMMSVFKVHQALALCNDFDNKGISLDTLVKIDRNRLDSKTWSPMMKDYSELVISLTVRDLLRYTIAQSDNNASNLMFKDMVNVAQTDSFIATLIPRSSFQIAYTEEEMSADHDRAYFNYTSPLGAAMLMNRLFTESIVSGEKQSFIKNTLKECVTGTDRIVAPLLDKERVSIAHKTGSGDVNENGILAAHNDVAYICLPNNVCYTLAIFVKDFKGNESQASQYVAHISEVVYSLLIQNSAIP
erm(F) macrolide resistance	442	fig|6666666.52503.peg.976	28325	27525	-	Dimethyladenosine transferase (E	FIG00000225	atgacaaaaaagaaattgcccgttcgttttacgggtcagcactttactattgataaagtgctaataaaagatgcaataagacaagcaaatataagtaatcaggatacggtttttagatattggggcaggcaaggggtttcttactgttcatttattaaaaatcgccaacaatgttgttgctattgaaaacgacacagctttggttgaacatttacgaaaattattttctgatgcccgaaatgttcaagttgtcggttgtgattttaggaattttgcagttccgaaatttcctttcaaagtggtgtcaaatattccttatggcattacttccgatattttcaaaatcctgatgtttgagagtcttggaaattttctgggaggttccattgtccttcagttagaacctacacaaaagttattttcgaggaagctttacaatccatataccgttttctatcatactttttttgatttgaaacttgtctatgaggtaggtcctgaaagtttctttccaccgccaactgtcaaatcagccctgttaaacattaaaagaaaacagttattttttgattttaagtttaaagccaaatacttagcatttatttcctgtctgttagagaaacctgatttatctgtaaaaacagctttaaagtcgattttcaggaaaagtcaggtcaggtcaatttcggaaaaattcggtttaaaccttaatgctcaaattgtttgtttgtctccaagtcaatgggtaaactgtttttttggaaatgctggaagttgtccctgaaaaatttcatccttcgtag	MTKKKLPVRFTGQHFTIDKVLIKDAIRQANISNQDTVLDIGAGKGFLTVHLLKIANNVVAIENDTALVEHLRKLFSDARNVQVVGCDFRNFAVPKFPFKVVSNIPYGITSDIFKILMFESLGNFLGGSIVLQLEPTQKLFSRKLYNPYTVFYHTFFDLKLVYEVGPESFFPPPTVKSALLNIKRKQLFFDFKFKAKYLAFISCLLEKPDLSVKTALKSIFRKSQVRSISEKFGLNLNAQIVCLSPSQWVNCFLEMLEVVPEKFHPS

Table 3.
AniB Values for additional multi-drug resistant isolates

	Bacteroides fragilis 638R	*Bacteroides fragilis* YCH46	*Bacteroides fragilis* NCTC 9343	*Bacteroides fragilis* HMW 615	Clinical Isolate	*Bacteroides fragilis* HMW 610	*Bacteroides fragilis* HMW 616
Bacteroides fragilis 638R	**100***	**98.56**	**98.58**	**98.3**	86.63	86.58	87.23
Bacteroides fragilis YCH46		**100**	**98.59**	**98.61**	86.64	86.59	87.25
Bacteroides fragilis NCTC 9343			**100**	**98.18**	86.64	86.79	86.93
Bacteroides fragilis HMW 615				**100**	86.17	86.55	87.2
Clinical Isolate					**100**	**98**	**96.66**
Bacteroides fragilis HMW 610						**100**	**97.56**
Bacteroides fragilis HMW 616							**100**

*bold indicates ANIb value above 95%

Susceptibility Testing

A 0.5 McFarland suspension of organism was made in sterile saline and immediately inoculated to Brucella agar with 5% sheep blood, hemin and vitamin K (Remel, Lenexa, KS, USA). The *B. fragilis* ATCC 25285 strain was used for quality control purposes. E-tests corresponding to the antimicrobial drugs shown in the text Table were used in accordance with the manufacturer's instructions (bioMérieux, Marcy l'Etoile, France). All plates were incubated under anaerobic conditions for 42–48 hours before reading. Where available, antimicrobial susceptibility test results were interpreted according to the Clinical Laboratory Standards Institute (CLSI) MIC interpretive standards for anaerobes. Resistance to metronidazole and imipenem was confirmed by macrobroth dilution using Brucella broth supplemented with hemin (5 µg/mL), vitamin K1 (1 µg/mL) and lyse horse blood (5% v/v). Doubling dilutions of metronidazole and imipenem over a final concentration range of 0.05–64 µg/mL were tested and the tubes incubated anaerobically for 48 hours before reading.

Reference

1. Richter M, Rossello-Mora R. Shifting the genomic gold standard for the prokaryotic species definition. Proc Natl Acad Sci U S A. 2009;106:19126–31. PubMed http://dx.doi.org/10.1073/pnas.0906412106
